# Valuing Mobile Health: An Open-Ended Contingent Valuation Survey of a National Digital Health Program

**DOI:** 10.2196/mhealth.9990

**Published:** 2019-01-17

**Authors:** Camilla Somers, Eleanor Grieve, Marilyn Lennon, Matt-Mouley Bouamrane, Frances S Mair, Emma McIntosh

**Affiliations:** 1 Health Economics and Health Technology Assessment University of Glasgow Glasgow United Kingdom; 2 Digital Health and Wellness Group Department of Computer and Information Sciences University of Strathclyde Glasgow United Kingdom; 3 General Practice and Primary Care University of Glasgow Glasgow United Kingdom

**Keywords:** mHealth, public health, delivery of health care, public health systems research

## Abstract

**Background:**

Changing population demographics and technology developments have resulted in growing interest in the potential of consumer-facing digital health. In the United Kingdom, a £37 million (US $49 million) national digital health program *delivering assisted living lifestyles at scale* (dallas) aimed to deploy such technologies at scale. However, little is known about how consumers *value* such digital health opportunities.

**Objective:**

This study explored consumers’ perspectives on the potential value of digital health technologies, particularly mobile health (mHealth), to promote well-being by examining their *willingness-to-pay* (WTP) for such health solutions.

**Methods:**

A contingent valuation study involving a UK-wide survey that asked participants to report open-ended absolute and marginal WTP or willingness-to-accept for the gain or loss of a hypothetical mHealth app, *Healthy Connections*.

**Results:**

A UK-representative cohort (n=1697) and a dallas-like (representative of dallas intervention communities) cohort (n=305) were surveyed. Positive absolute and marginal WTP valuations of the app were identified across both cohorts (absolute WTP: UK-representative cohort £196 or US $258 and dallas-like cohort £162 or US $214; marginal WTP: UK-representative cohort £160 or US $211 and dallas-like cohort £151 or US $199). Among both cohorts, there was a high prevalence of zeros for both the absolute WTP (UK-representative cohort: 467/1697, 27.52% and dallas-like cohort: 95/305, 31.15%) and marginal WTP (UK-representative cohort: 487/1697, 28.70% and dallas-like cohort: 99/305, 32.5%). In both cohorts, better general health, previous amount spent on health apps (UK-representative cohort 0.64, 95% CI 0.27 to 1.01; dallas-like cohort: 1.27, 95% CI 0.32 to 2.23), and age had a significant (*P*>.00) association with WTP (UK-representative cohort: −0.1, 95% CI −0.02 to −0.01; dallas-like cohort: −0.02, 95% CI −0.03 to −0.01), with younger participants willing to pay more for the app. In the UK-representative cohort, as expected, higher WTP was positively associated with income up to £30,000 or US $39,642 (0.21, 95% CI 0.14 to 0.4) and increased spending on existing phone and internet services (0.52, 95% CI 0.30 to 0.74). The amount spent on existing health apps was shown to be a positive indicator of WTP across cohorts, although the effect was marginal (UK-representative cohort 0.01, 95% CI 0.01 to 0.01; dallas-like cohort 0.01, 95% CI 0.01 to 0.02).

**Conclusions:**

This study demonstrates that consumers value mHealth solutions that promote well-being, social connectivity, and health care control, but it is not universally embraced. For mHealth to achieve its potential, apps need to be tailored to user accessibility and health needs, and more understanding of what hinders frequent users of digital technologies and those with long-term conditions is required. This novel application of WTP in a digital health context demonstrates an economic argument for investing in upskilling the population to promote access and expedite uptake and utilization of such digital health and well-being apps.

## Introduction

### Background

Globally, more than 50% of the world population owns a mobile device, rising to nearly 90% in the developed world [1]. Digital technology use is becoming integrated into our daily lives and clearly has potential to promote physical and psychological well-being [2]. Digital health is seen as having the potential to transform health care [3] at a time when changing population demographics and rising levels of chronic illness and multimorbidity (the presence of 2 or more long-term conditions) make change imperative [4]. However, this opportunity presents a number of challenges as developers must tackle a current underuse of readily available digital health innovations and there is a need for more evidence to aid understanding of *what* is of value to users [5]. In recent years, the United Kingdom has prioritized developing a digital health strategy to be implemented nationally [3,6]. A key driving force behind digital health is the need to move to more cost-effective health care delivery models, with the National Institute for Health and Care Excellence (NICE) announcing plans to develop a new digital health apps evaluation system to respond to the recent growth in digital health. Digital health, particularly mobile health (mHealth), refers to *raising awareness of health information via mobile or wireless devices* and has the potential to provide an alternative, less resource-intensive delivery of health to a changing population [7,8]. In their most recent communications report, Ofcom declared the United Kingdom to be a *smartphone society,* with more than 60% of the population owning a smartphone [9]. The number of mHealth apps continues to grow at an ever-increasing pace, with as many as 325,000 health apps available in 2017 and 78,000 new health apps added to major app stores in the last year [10]. However, existing studies have demonstrated the complex and highly variable nature of implementing successful well-being digital technologies and tools [11-15].

mHealth technology should be flexible and accessible for both users and practitioners [7]. Using digital devices also enables users to create platforms for support and self-management, providing opportunities for wider aspects of one’s well-being to be improved. Looking beyond health to include nonhealth aspects of quality of life, such as one’s sense of empowerment and ability to participate in community activities, has increasingly become a focal point of health service interventions [16,17]. Furthermore, NICE has emphasized the need for successful community engagement initiatives by health services to produce positive health gains and tackle health inequalities [18,19]. However, the personalization of digital health technologies and their focus on seeking to improve multiple broader aspects of health pose a number of challenges for economic evaluations in determining the value of their delivery and outcomes [20]. Issues include the need for wider measurement of costs and benefits as well as the handling of development costs [21].

In this paper, we present a contingent valuation (CV) willingness-to-pay (WTP) study for a hypothetical mHealth app that would deliver against 6 well-being outcomes alongside any other health services or treatments. The study examines the value the public places on improving broader well-being outcomes with mHealth. This was part of a wider program *delivering assisted living lifestyles at scale* (dallas). The dallas program launched in 2012 and funded by Innovate UK established 4 multiagency communities across the United Kingdom, who were to show “how independent living technologies, services and systems can be used to promote wellbeing, and provide integrated top quality health and care, enabling people to live independently” [22]. These communities worked in collaboration with a number of stakeholders, including health care services, industry, third sector voluntary organizations, and academic and government bodies, to explore how digital health can be delivered successfully for preventative care and to promote well-being across the United Kingdom [14]. Further details of the communities and their associated partnerships have previously been reported [14,23].

### Existing Studies

Research on WTP for specific treatment or disease management using digital health technologies has been conducted but is still in its infancy. In Ireland, they have examined women’s valuation of an integrated app and stand-alone app for postoperative monitoring post cesarean section [24]. WTP levels were considerably smaller than anticipated, and this was attributed to the participant’s experiences of paying small amounts for mobile phone apps previously [24]. In Bangladesh, a country where health care is provided on a fee for service basis, WTP for mobile phone short message service text messaging to promote diabetes self-management was explored [25]. The researchers found that participants were generally willing to pay for the service and that those males with higher household income and higher levels of education reported higher WTP levels. However, research on WTP for mHealth apps looking at improving broader lifestyle well-being outcomes is currently an understudied area. This study seeks to build on previous studies such as that by Callan and O’Shea [18], which focused on determining societal values for different telecare solutions for older people. Their study demonstrates that there is a preference for developing supportive technologies, which seek to keep older people in their community, and that above telecare for physical or cognitive care needs, strongest preferences were for telecare that sought to improve user’s social connections. This potential for mHealth as an individual’s own *tailored* health service is further emphasized by researchers such as Klasnja and Pratt [24] who argue that if delivered in a sensitive and appropriate manner, mHealth could be effective in managing both specific diseases and general health while also enabling communities to support one another. This would allow virtual networks or communities of users with similar goals or location to connect to one another [26]. Reviews of mHealth interventions have demonstrated that few evaluations have captured data that allow for consideration of economic outcomes and overall effectiveness and cost-effectiveness of interventions [27-31]. The lack of standardization in the delivery of mHealth programs means that currently full societal outcomes are not being captured and decision makers cannot make fully informed decisions when comparing the cost-effectiveness of different programs [32]. The potential to understand the value the public places on aspects of broader well-being, lifestyle, or other measures of individual autonomy is important and a much-needed advance in evaluations of these types of person-centered digital health and wellness products and services. Indeed, guidelines for producing high-quality evidence of digital health programs have emphasized the need for appropriate analytical methodology that can capture these noncost-related outcomes [30,33].

### Broader Lifestyle Outcomes (The 6Cs)

As the name of dallas explicitly highlights, the program from the outset had an emphasis on making a positive impact on citizens’ *lifestyles*, moving away from a purely medical model. As the dallas communities’ implementation plans included specific targets on recruitment number, the program funder, Innovate UK, took steps to ensure that impact on lifestyles could also be objectively measured as part of the broader dallas initiatives’ deployment. For this purpose, it proposed the use of 6 key concepts that could demonstrate commonality of purpose for the broader program regardless of the details of each of the many communities’ interventions. These key concepts were called the *6Cs*, namely, connectedness, control, choice, collaboration, community, and contribution. To achieve some degree of consensus on how these key concepts could be applied to each community implementation plan, a workshop was organized at the outset of the program by Innovate UK in June 2012 (Birmingham). Key representatives from the program funder, the 4 dallas communities, and the program evaluation team (ie, University of Glasgow) attended this workshop. During the workshop, a series of focus groups was undertaken to develop and iteratively refine a detailed mapping of how the 6Cs applied to each community’s specific implementation plan [13,14,34]. Table 1 [22] also demonstrates how mHealth app features could foster and improve a user’s sense of each of the 6Cs.

**Table 1:** Innovate UK 6Cs

Concept	Definition	Example of concept as a mobile health app feature
Connectedness	Connections and networking between individuals through real or virtual interaction	Call and messaging features to connect directly to friends or family, local health care, and other users with similar health conditions or goals in their network
Control	Individuals’ ability to control their own health care and well-being	Ability to personalize profile and create health goals and details of their health status. Can control who can see aspects of their health status they wish to share and reflect on what is happening in their lives
Choice	Choice in terms of products, services, and systems available to suit needs	Being provided with a suite of alternative apps to manage symptoms at home
Collaboration	Organizations and communities collaborating together to develop and deliver products, systems, and services	Can share health data with others and contribute to forums to raise issues and share experiences
Community	Individuals that are part of a community rather than living in isolation, connected to others with shared needs, interests, and aims	Can share to and link with Web-based and local communities through social media and can gain information about local community resources that might be helpful for individuals or their caregivers
Contribution	Individuals’ ability to contribute to their local community	By selecting their *home* location and their interest areas, individuals can receive alerts about local happenings and can also organize their own events or groups

## Methods

### Contingent Valuation

CV is a form of stated preference methodology used to estimate welfare gains or losses. CV allows researchers to value nonmarket commodities [35]. In the absence of a market for a good, such as that occurring in publicly funded health care systems, surveys can be used to directly ask participants to report their WTP or willingness-to-accept (WTA) the gain or loss of a hypothetical good or service. Values elicited are then regarded as a value indicator and measure of the demand for the good [36]. This allows a direct valuation for the 6Cs, which could be used within a cost-benefit analysis (CBA). The application of WTP methodology can provide insights into what people value (or not) in future digital health services and, therefore, inform both commercial endeavors to provide what the market wants and will pay for and also the planning of health and care services in a future where health care will most certainly be supported by digital products. In this study, the approach provides an indication of people’s valuation of a change in the 6Cs.

The study design was a self-complete, stated preference, open-ended WTP survey embedded within a questionnaire, which also asked respondents to self-report sociodemographic information, their general health status, and details of any existing health conditions as well as report their current app- and digital- device and services ownership and usage. Data were collected through the use of Web-based survey panels accessed through the survey host, ResearchNow. In exchange for completing surveys, members were offered e-currency (points). For a 10 minute survey, they receive approximately £0.50 (US $0.66). Panelists accrue this as e-currency and can exchange it for goods.

### Sample

Data were collected from 2 cohorts of participants. First, ResearchNow contacted UK-based panel members to create a representative sample based on age, gender, and income demographics. Second, a subsample whose characteristics mirror those of dallas communities (a *dallas-like* sample) gave the opportunity to generate a WTP estimation for those citizens currently being targeted by dallas and similar National Health Service (NHS) initiatives.

Following guidance on optimal sample sizes for CV open-ended questions, it was predicted that a sample size of no less than 400 was required [37]. To undertake subgroup analyses by including a cohort *dallas-like* sample and to take into consideration the prevalence of multimorbidity in the UK population, advice was sought from a statistician and existing literature and the sample size was increased to approximately 2000 [4,38].

### Survey Design

#### Contextual Information

Pivotal to the success and accuracy of valuations derived from CV studies is the development of realistic, plausible scenarios, which are then presented to individuals. Poorly designed, cognitively burdensome surveys, which respondents find unrealistic, can generate biased responses and can undermine the reliability of their WTP or WTA estimates [35,39]. Before completing the WTP task, respondents were presented with contextual information (see Multimedia Appendix 1).

Respondents were then presented with a hypothetical mHealth app called *Healthy Connections* that was designed to describe the broader lifestyle and well-being outcomes (the 6Cs) that were embedded as part of the dallas program, as described in Table 1.

#### Willingness-to-Pay Questions

A key consideration of a stated preference WTP study is the type of hypothetical payment *vehicle* used to generate monetary values. The payment vehicle must be realistic to avoid provoking a rejection of the task [40]. For the purpose of this study, a monthly subscription fee was used. Both absolute and marginal WTP questions were included [41]. An open-ended WTP question confirmed the participants’ absolute WTP for access to the app and their marginal WTP. The absolute WTP question was framed with participants asked to consider their WTP in relation to what they currently pay to stay connected to others (ie, mobile broadband charges) and for health benefits (ie, mHealth apps or gym memberships). This was to ensure that WTP for the physical mHealth features was similar to that for the current mHealth and health service markets. However, the research team acknowledged that such framing could introduce bias into the WTP results by asking respondents to state a WTP linked to their current spending on similar health or digital services and would not fully capture the respondents’ valuation of the health benefits of an improvement in their sense of the 6Cs. Therefore, the marginal WTP question asked participants to consider the maximum they would be willing to pay for improved levels of 6Cs from their current 6Cs’ situation. Capturing both these results allows for the researchers to understand the value placed on the health improvement expected and the value for the product or service needed to produce these.

#### Sociodemographic and Economic Characteristics

Our hypothesis was that general health (complete physical, mental, and social well-being) and experiences of living with long-term health conditions were likely to affect valuations for the 6Cs and that participants’ familiarity with mobile technology and mHealth apps may lead to a higher WTP for the *Healthy Connections* app. Respondents were asked to rate their overall general health and well-being from *excellent* to *poor*. When referring to long-term conditions, the examples of asthma, diabetes, cancer, psoriasis, lung disease, heart disease, and depression were provided to respondents to demonstrate the diversity of conditions they should consider when describing their own health. In addition, we hypothesized that younger users could have a higher WTP (more risk taking and more familiar with newer technologies); however, we acknowledged that this had to be balanced with the likelihood that their incomes will likely be lower. Finally, we expected an income effect, with those with higher incomes and with more disposable income reporting a higher WTP.

To examine these possible influences, questions on health (self-reported general health, long-term health conditions, and medication history); ownership of, and accessibility to, technologies (computers, smartphones, internet, previous health apps’ history, and total monthly spending on technology); age; and total annual income were included in the survey.

### Validity Testing: Pilot Survey

To test the face validity of the survey and the suitability of the open-ended question format, a soft pilot survey was conducted (n=52) before the main Web-based survey. From these results, we were able to test the validity of our survey and whether our open-ended WTP question format was suitable and understood by participants. No respondents were reported to have struggled with the task or were unable to complete.

### Analysis

Stata/12SE software (Stata Corp)was used to analyze the data [42]. To estimate a demand function for the 6Cs and the mean WTP, linear regression analyses were used. The open-ended WTP was used as the continuous, dependent variable. Socioeconomic characteristics of the participants were used as *predictor* independent variables. This allowed for the opportunity to test and profile WTP. Furthermore, the pilot study data demonstrated the wide range of WTP responses and prevalence of zero responses. Zero valuations are common in this form of study as the good or service in question is a UK health app and would be part of the suite of NHS services, which are all free at point of use (covered by taxation), and thus, there would be an assumption that this mHealth app should not differ. Indeed, all apps on the NHS digital library are free for use. Thus, to reduce the large skew in the results and learning from the pilot study, the WTP values were converted into natural logarithms (LN) before running regressions with the main survey data. It should be noted that before taking the natural log, a value of 1 was added to WTP values to avoid the problem of 0 values. Thus, in each of the models presented, the dependent variable used was LN(WTP)=log(1+WTP). The same calculation was conducted for the marginal WTP values.

### Ethics Approval and Consent to Participate

The survey and project received confirmation of University of Glasgow ethics approval (July 29, 2015).

## Results

### Survey Cohorts

Throughout September to October 2015, a total of 2002 respondents were surveyed for the 2 cohorts (UK general population and *dallas-like* cohorts). The general UK-representative cohort consisted of 1697 respondents. On the basis of the UK general population, 48.67% (826/1697) of the cohort were male and 51.33% (871/1697) were female. The average age of respondents was 47 years, ranging from 18 to 89 years. The majority of respondents (1421/1697, 83.73%) were from the United Kingdom. Furthermore, 68.18% (1157/1697) of the sample were in a relationship, whereas 61.52% (1044/1697) had children. Moreover, 18.70% (317/1697) of respondents had a total household income of less than £14,999 (US $19,820), 60.00% (1017/1697) earned £15,000 to £49,999 (US $19,821 to US $66,069), and the remaining 21.40% (363/1697) earned more than £50,000 (US $66,070). In addition, 62.90% (1066/1697) had no long-term health conditions, whereas 37.20% (631/1697) had long-term health conditions. In total, 14.50% (246/1697) were smokers, and 47.55% (807/1697) stated they took medications regularly. The dallas-like cohort consisted of 305 respondents. Of 305 respondents, 27.9% (85/305) were male and 72.1% (220/305) were female. The cohort had an average age of 48 years, with an age range of 16 to 86 years. Similar to the UK general population cohort, 67.2% (205/305) were in a relationship and 63.3% (193/305) had children. Overall, 15.1% (46/305) of respondents had a total household income of less than £14,999 (US $19,820), 60.0% (183/305) earned £15,000 to £49,999 (US $19,821 to US $66,069), and the remaining 25.0% (76/305) earned more than £50,000 (US $66,070). Moreover, 65.2% (199/305) had no long-term health conditions, whereas 34.8% (106/305) had long-term health conditions. In addition, 14.4% (44/305) of the cohort were smokers, and 43.6% (133/305) took medications regularly. The full details of the 2 cohorts can be found in Multimedia Appendix 2.

### Absolute and Marginal Willingness-to-Pay

Summary statistics of both cohorts’ respondents’ absolute WTP and marginal WTP are shown in Table 2. When compared with the WTP figures of the UK general population sample, *dallas-like* respondents reported lower mean WTP and marginal WTP than that estimated in the general population survey, whereas both samples’ marginal WTP estimates had a range of £600 (US $793). Furthermore, among both cohorts, there was a high prevalence of zeros for both the absolute WTP (UK general population sample: 467/1697, 27.52% and dallas-like cohort: 95/305, 31.15%) and marginal WTP (UK-representative cohort: 487/1697, 28.70% and dallas-like cohort: 99/305, 32.5%) estimates.

Results of linear regressions conducted are shown in Table 3. The results illustrate that for the general UK population cohort, respondents who felt they *disagree*, were *neutral,* or *agree* to the statement that they feel connected to health care providers were more likely to pay more (*P*<.05) for the *optimal* scenario presented to them than the reference group (*strongly disagree*). Furthermore, feeling connected to social care services or providers was shown to act as a predictor of higher WTP. The dallas-like cohort demonstrated that the only potential predictor was the sense of *control* responses. Higher levels of control over health management acted as an inverse indicator of WTP as respondents (relative to the reference level of *strongly disagree*) were more likely to pay less for the improvement provided by *Healthy Connections*.

**Table 2:** Absolute and marginal willingness-to-pay.

Descriptive statistics	General UK population (n=1697)		Dallas-like respondents (n=305)	
	Absolute WTP^a^ (£/month)	Marginal WTP (£/month)	Absolute WTP (£/month)	Marginal WTP (£/month)
Mean	16.3 (US $21.5)	13.3 (US $17.6)	13.5 (US $17.8)	12.6 (US $16.7)
Median	5 (US $6.6)	5 (US $6.6)	5 (US $6.6)	5 (US $6.6)
Mode	0	0	0	0
Range	£0-900 (US $0-1189)	£0-600 (US $0-793)	£0-600 (US$0-793)	£0-600 (US $0-793)

^a^WTP: willingness-to-pay.

**Table 3:** Linear regression results for marginal willingness-to-pay and respondents’ current 6Cs levels (adjusted for age, total household income, and gender).

Variable			General UK population (n=1697)			Dallas-like cohort (n=305)		
			Coefficient	*P* value	95% CI	Coefficient	*P* value	95% CI
**Connections (I feel connected with/to...)**								
	**My friends and family**							
		Strongly disagree	—^a^	—	—	—	—	—
		Disagree	−0.2	.59	−0.82 to 0.47	−0.16	.85	−1.79 to 1.48
		Neutral	−0.29	.34	−0.89 to 0.30	−0.44	.53	−1.79 to 0.92
		Agree	−0.36	.22	−0.94 to 0.21	−0.44	.52	−1.79 to 0.91
		Strongly agree	−0.36	.22	−0.94 to 0.22	−0.47	.5	−1.83 to 0.89
	**Health care services and/or providers**							
		Strongly disagree	—	—	—	—	—	—
		Disagree	0.46	.05	0.00 to 0.93	0.33	.59	−0.88 to 1.54
		Neutral	0.44	.06	−0.01 to 0.88	0.19	.75	−0.98 to 1.36
		Agree	0.46	.05	0.01 to 0.92	0.06	.93	−1.14 to 1.26
		Strongly agree	0.42	.1	−0.09 to 0.92	0	1	−1.40 to 1.41
	**Social care services and/or providers**							
		Strongly disagree	—	—	—	—	—	—
		Disagree	0.06	.59	−0.17 to 0.30	−0.06	.82	−0.60 to 0.48
		Neutral	0.3	.01	0.08 to 0.53	0.36	.16	−0.15 to 0.87
		Agree	0.89	0	0.61 to 1.17	0.5	.14	−0.16 to 1.16
		Strongly agree	0.98	0	0.55 to 1.42	0.77	.2	−0.42 to 1.95
**I feel I make a contribution in my community**								
	Strongly disagree		—	—	—	—	—	—
	Disagree		−0.08	.68	−0.45 to 0.29	0.11	.85	−1.00 to 1.22
	Neutral		0.1	.59	−0.27 to 0.47	0.22	.69	−0.87 to 1.32
	Agree		0.2	.31	−0.19 to 0.58	0.45	.44	−0.69 to 1.58
	Strongly agree		0.17	.45	−0.27 to 0.61	−0.06	.93	−1.35 to 1.23
**I feel I have control in how I manage my health and well-being**								
	Strongly disagree		—	—	—	—	—	—
	Disagree		−0.06	.89	−0.83 to 0.72	−1.67	.11	−3.70 to 0.36
	Neutral		0.06	.88	−0.70 to 0.81	−2.48	.01	−4.31 to −0.65
	Agree		−0.19	.62	−0.94 to 0.56	−2.62	.01	−4.44 to −0.79
	Strongly agree		−0.32	.41	−1.10 to −0.45	−2.35	.01	−4.22 to −0.47
**I feel I have a choice in how I manage my health and well-being**								
	Strongly disagree		—	—	—	—	—	—
	Disagree		0.44	.25	−0.32 to 1.19	0.86	.44	−1.35 to 3.08
	Neutral		0.42	.27	−0.33 to 1.17	1.39	.2	−0.73 to 3.51
	Agree		0.61	.11	−0.13 to 1.35	1.88	.08	−0.24 to 4.00
	Strongly agree		0.65	.09	−0.11 to 1.41	1.64	.13	−0.47 to 3.74
**I feel that I am part of my community**								
	Strongly disagree		—	—	—	—	—	—
	Disagree		0.06	.78	−0.34 to 0.46	0.24	.7	−0.99 to 1.46
	Neutral		0.04	.86	−0.36 to 0.43	0.32	.59	−0.85 to 1.49
	Agree		0.24	.25	−0.17 to 0.65	0.55	.37	−0.65 to 1.76
	Strongly agree		0.24	.31	−0.23 to 0.72	1.4	.06	−0.03 to 2.82

^a^Standard linear regression conducted and, therefore, coefficients show the difference between the variable category and “Strongly Disagree” as reference category. Strongly disagree *P* values are not applicable.

### Sociodemographic and Economic Characteristics

Both cohorts indicated that respondents’ age has a significant (*P*<.05) relationship with WTP (UK population cohort: −0.1, 95% CI −0.02 to −0.01; dallas-like cohort: −0.02, 95% CI −0.03 to −0.01), illustrating that younger respondents will pay more for the health connections app. In the general UK population cohort, relative to the reference level group (≤£14,999/US $19,819), £15,000 to £29,999 (US $19,821 to $39,641), income level acts as a significant, positive predictor of higher WTP (0.21, 95% CI 0.14 to 0.4). This is the theoretically expected result. However, this trend is not shown in the income earning brackets of £30,000 to £49,999 (US $39,642 to $66,069) or ≥£50,000 (≥US $66,070), and no relationship between income and WTP is estimated in the dallas-like cohort. Gender differences were statistically significant only in the dallas-like cohort where females had a lower WTP relative to the male reference level (−0.35, 95% CI −0.69 to −0.01). For both cohorts, general health was a positive predictor of WTP, with those respondents who describe themselves in better health being more likely to spend more for the *Healthy Connections* app.

However, only in the general UK population sample, there was a statistically significant positive relationship between regularly taking medication and higher WTP (0.16, 95% CI −0.01 to 0.32). This trend was not statistically significant in the dallas-like cohort, and neither cohort illustrated that long-term illness was a factor influencing WTP. These results suggest that individuals who are currently in better health value the mHealth app the most. The full analysis can be found in Multimedia Appendix 3.

In the general UK population cohort, higher WTP values were positively associated with current total monthly payments on phone, internet, and additional features (ie, app subscriptions), with respondents who reported they currently spent more on these services monthly stating larger WTP for the *Healthy Connections* app. Respondents who described themselves as having the internet yet never use it had a significant (*P*<.05), positive relationship with WTP (1.18, 95% CI 0.34 to 2.01) and were more likely to pay a higher amount than the reference group who have no access to the internet at home. In addition, those who have access to the internet at home and use it regularly demonstrated a negative association with WTP (−0.5, 95% CI −0.93 to −0.07) relative to the reference group. Finally, owning a computer but rarely using it acted as a statistically significant predictor of an inverse WTP (−0.5, 95% CI −0.95 to −0.05), paying less than those who do not own a computer. Results from the dallas-like cohort highlighted that owning a computer or smartphone, having regular access to the internet, and the total monthly payment for phones and internet usage (and additional features) were not indicators for higher WTP. For both cohorts, previous amount spent on health apps acted as a significant positive predictor of WTP, yet the effect was minimal (0.01). These linear regression results on familiarity and accessibility to mHealth and technology demonstrate that aside from the UK population cohort’s positive association between current payments for phone, internet, and additional features and higher WTP, having access to a computer and internet is not a clear indicator of higher value and WTP for mHealth and was shown to be a negative indicator of WTP in the UK population cohort (see Multimedia Appendix 3 for further details).

## Discussion

### Principal Findings

Drawing on data from 2 cohorts, we have demonstrated that both the general UK population and a cohort whose characteristics are similar to those already receiving a large-scale digital health program valued both the access to improved broader well-being (6Cs) and the development of an mHealth app such as *Healthy Connections*. This WTP study revealed a positive valuation of the 6Cs of £196 (US $258) per annum for the general UK population cohort’s absolute WTP values and a value of £160 (US $211) for the marginal WTP (ie, to move participants from their current 6Cs’ position to the highest level of 6Cs). In addition, the dallas-like cohort’s absolute WTP valued the 6Cs mHealth app at £162 (US $214) and a value of £151 (US $200) for the marginal WTP. By incorporating questions about both these forms of WTP, we were able to evidence positive valuations for both the possibility of the improvement in their sense of each of the 6Cs’ lifestyle components from their current 6Cs’ experience (marginal WTP) and also for the value for the app itself (absolute WTP). Therefore, the study’s results lend themselves to a wider evidence base than just mHealth apps and solutions and can demonstrate that investment in other activities or services, which seek to foster improvement in 6Cs lifestyle components, may also be a worthwhile investment in resource allocation.

Furthermore, the study illustrates that for the general UK population cohort, this WTP was positively affected by participants’ existing sense of connection to social care services and having current connections to health care services or staff. Conversely, dallas-like respondents who felt they already had a sense of control in their health and well-being management demonstrated an inverse relationship to WTP. Such sensitivity to individual needs and preferences may represent a costly or time-consuming development process, yet these results further evidence the challenges associated with obtaining consistent, homogenous preferences from WTP surveys of digital health programs.

In addition, the valuations are based on the understanding that the *Healthy Connections* mHealth app was a generalizable (not disease specific) mHealth service suitable for the whole population. The research team envisaged that the 6Cs lifestyle components were aspects of health and well-being that could be valuable for all users, not just those currently suffering from an illness. The results highlighted that for both cohorts, better self-reported health was positively associated with WTP, and long-term illness was not a factor that influenced respondents’ WTP, whereas regular medication was associated with higher WTP in the general UK population cohort. The lack of clarity on the relationship between a person’s health, health behaviors, and WTP for an app such as *Healthy Connections* in the results suggest that the true value of an app such as *Healthy Connections* could be investigated further with a more detailed focus on types of health (physical and mental well-being) and disease types. The strength of this study is that it shows there is an inherent value for the 6Cs for multiple types of users with differing health needs and status and, thus, provides initial evidence of the need for further investigation of the role of mHealth to improve lifestyle. Examination of sociodemographic and economic factors and familiarity with mHealth technology demonstrated some user traits that may help inform future development of similar mHealth apps. Age was shown to act as a predictor of higher WTP in both cohorts, with younger respondents being willing to pay more for the app. Female respondents were shown to have a lower WTP than their male counterparts in the dallas-like cohort. Beyond higher current spending on digital devices and health apps as indicators of higher WTP, no clear trends were shown across internet, computer, or smartphone access and use. In fact, although the dallas-like cohort results showed no statistically significant trends, the general UK population results showed owning a home computer and using it rarely and being regular users of internet as negatively associated with WTP. This variability in the results highlights a clear need for more research on how *type* of digital platform or accessibility options may impact the success of mHealth apps and the investment in upskilling of users required. Importantly, it suggests that it is incorrect to assume that levels of access to smartphones or the internet can be used to reliably predict uptake of digital health services. Surprisingly, despite other cost indicators acting as indicators of WTP, an increase in total household income was not shown to have the expected significant trend on WTP. There was an increase in WTP, relative to the reference level of *less than £14,999* (US $19,819); however, this was not significant beyond £15,000 to 29,999 (US $19,821 to $39,641). Such confounders suggest that although our study demonstrates that there is clear evidence to support the rationale for developing mHealth as a new supporting method for health care delivery, inherently their use or appropriateness may not be solely reliant on income but perhaps existing familiarity and acceptance for these forms of health-related technologies as a norm or part of daily routine.

### Limitations

A limitation of the dallas evaluation is that impact on health and social care resource use was not captured. We can, however, compare our WTP results with both the cost of a dallas-type product and also the cost of the dallas program. The cost of an app can range from free to £1, £10s, and £100s [43], dependent on the type of app. The dallas program included costs of recruiting and reaching users and interoperability costs (ie, enabling work to integrate the apps with health records and social care systems). Further research would enable these WTP results to be used in a CBA framework [20]. To do this, longer-term follow-up would be required to capture impact on health and social care resource use and any potential cost-savings, for example, an attributable reduction in hospital admissions in addition to the cost of an app itself.

The open-ended WTP approach is typically associated with large values, skewed data, and zeros [44]. We have found this to be the case in this study; however, through the decision to capture data on both absolute and marginal WTP, we were able to mitigate the effect of anchoring bias. The study was able to determine value of both the development of mHealth apps and of users’ improving their sense of the 6Cs [45].

Another limitation of this study is the UK context, an environment in which there is free universal access to health care. WTP might be quite different in a fee-paying environment, for example, the United States, where use of mHealth apps to avoid attending traditional health care professionals might be valued differently.

Researchers such as Klasnja and Pratt [24] have highlighted how advancements in mHealth technology could, if delivered in a sensitive and appropriate manner, not only be effective for solely specific disease management or general health improvement but could also leverage social networks and communities to support one another. This would allow virtual networks or communities of users with similar goals or location to connect to one another.

### Conclusions

This study demonstrates that although consumers value mHealth solutions that promote well-being, social connectivity, and health care control, mHealth is not universally embraced, and more research is needed to understand the relationship between health status of the potential user and how to tailor an app such as *Healthy Connections* to suit their needs. Furthermore, the study evidences that accessibility and use of smartphones, internet, or computers do not equate to WTP for mHealth apps. For mHealth to achieve its potential, apps need to be tailored to the accessibility and health needs of the user and more understanding of what hinders the use or acceptability of mHealth apps to even the most frequent users of multiple digital technologies is required. A key challenge is how to engage people with long-term conditions to encourage uptake of mHealth apps. This novel application of WTP in a digital health context presents a compelling economic argument for further research and future investment in both improving the accessibility and, where necessary, upskilling the population to promote access and expedite uptake and utilization of such digital health and well-being apps.

## Appendix

Multimedia Appendix 1 The willingness-to-pay survey used in the study.

Multimedia Appendix 2 Sociodemographic information about both cohorts.

Multimedia Appendix 3 Linear regression results of cohorts’ sociodemographic and economic characteristics and willingness-to-pay and familiarity and accessibility to mobile health technology and willingness-to-pay.

## Abbreviations

CBA: cost-benefit analysis
CV: contingent valuation
dallas: delivering assisted living lifestyles at scale
LN: natural logarithms
mHealth: mobile health
NHS: National Health Services
NICE: National Institute for Health and Care Excellence
WTA: willingness-to-accept
WTP: willingness-to-pay
