# Supplementary material for: Valuing Mobile Health: An Open-Ended Contingent Valuation Survey of a National Digital Health Program
Source: JMIR Mhealth Uhealth. 2019 Jan 17;7(1):e3. doi: 10.2196/mhealth.9990 (PMC6354197; doi:10.2196/mhealth.9990)
Supplement: Multimedia Appendix 1 [file mhealth_v7i1e3_app1.pdf]

Multimedia appendix A

Healthy Connections- Valuing mobile health

You are invited to participate in this survey exploring how much people value mobile health for improving health and wellbeing.

What is mHealth?

Mobile health (mHealth) refers to **raising awareness of health information via mobile and wireless devices such as mobile telephones and the Internet.** Today, the delivery of healthcare is drastically changing, with people gaining increased knowledge and awareness through the vast availability of information on the internet.

What is an 'App'?

Currently, the National Health Service (NHS) and some voluntary organisations are recommending the use of 'apps' to increase health awareness. An app is a program on a portable device which helps you to perform tasks. Not limited to just mobile phones, apps can be used on many devices including personal digital assistants (PDA), portable media players, (MP3/MP4 players), handheld video-game consoles and portable computers (tablets, smartbooks and ipads).

Apps and health

Apps are being increasingly recognised as an alternative means of providing help for users to manage long term health conditions and gain further information about maintaining a healthy lifestyle, keeping fit or even losing weight.

What does mHealth mean for me?

Apps allow users to incorporate managing their health into their daily routine with ease and at their own convenience. By having access to resources and advice whenever it suits them, users have more control over their health and it encourages users to feel empowered and more informed about their condition or general health and wellbeing.

Community-based healthcare

UK-wide the NHS is moving towards services which work with communities to deliver care that is more personalised. Providing services within patients' homes and in the local community helps encourage a more holistic and comfortable experience.

How does this link to mHealth apps?

Communities can play an essential role in successful healthcare. There is growing evidence that more socially active individuals who experience strong relationships with others often report better quality of life than those suffering from loneliness and social isolation. Indeed, loneliness is now recognised as an important health concern associated with reduced quality of life. Therefore, the ever-growing popularity of mobile technology, its application to numerous aspects of daily life, and its capacity to enable users to link and share experiences, thereby reducing social isolation, creates an ideal opportunity to further this community approach to healthcare delivery. The development of an app which helps people to discuss and share health experiences directly with others and healthcare providers should encourage individuals to take control of their own care and adopt a healthier lifestyle.

What specific aspects of my wellbeing can mHealth improve?

Through using mHealth and making it a widely used and familiar component of UK healthcare delivery, it is possible that you will feel much more **CONNECTED** to friends/family and health and social care providers through continuously being able to share information about your health and wellbeing with them. mHealth gives you more **CONTROL** and **CHOICE** over your health as you can monitor your progress whenever or wherever suits you. Using the many features of apps, mHealth helps you **COLLABORATE** with others in making decisions about your health and wellbeing. Lastly, through personalising how you use, receive and share information and experiences through the apps, you can feel more a part of your **COMMUNITY** and chose to **CONTRIBUTE** to it at your own convenience.

What is the aim of this survey?

We are interested in finding out how much people value mHealth apps. In this survey you will be presented with an imaginary mHealth app called ***'Healthy Connections'*** that seeks to promote users' social connections as a means of improving your health. The app is for those interested in taking control of their health, whether battling long-term conditions or just

seeking improved health. Healthy Connections provides a smart and personalised space for your health management. Healthy Connections is not just for use on your smartphone, but can also be downloaded onto portable computers for your convenience.

Healthy Connections: Imagine improving your health using an App

Please imagine that '*Healthy Connections'* is a new app soon to be launched, designed to help you gain control of your health all in one simple app. Users access a suite of six features allowing them to record their current health status, share their experiences with healthcare professionals, friends and family and receive updates of happenings in their local area.

6 Features of Healthy Connections

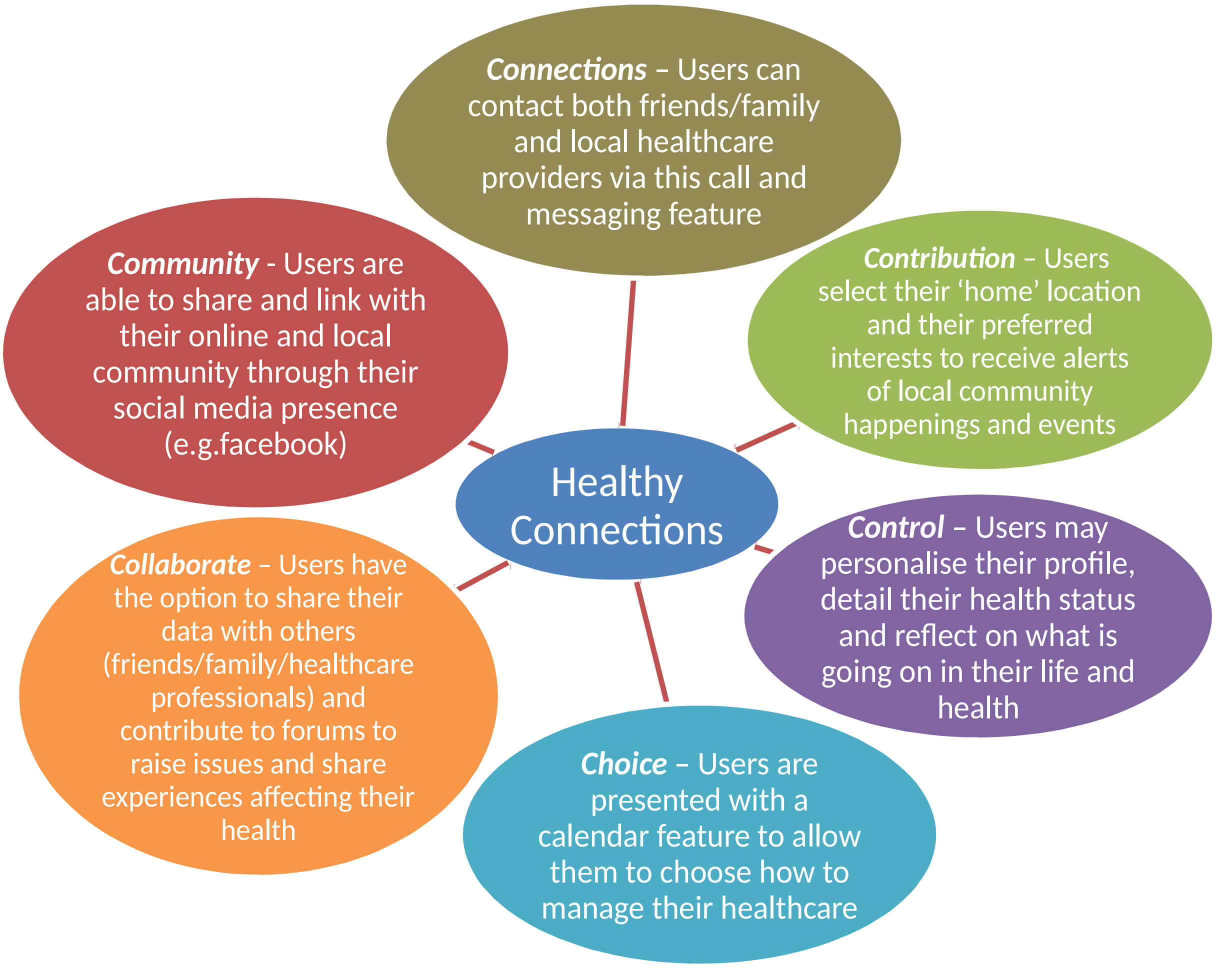

Rate your 'healthy connections'

Think about your current 'connection' circumstances and state of health and wellbeing. For each of the statements below, please **tick the box** that best describes your current lifestyle state today.

**STATEMENTS**	Strongly Disagree	Disagree	Neutral	Agree	Strongly Agree
I feel ***connected*** with/to…					
My friends and family	☐	☐	☐	☐	☐
Health care services and/or providers	☐	☐	☐	☐	☐
Social care services and/or providers	☐	☐	☐	☐	☐
I feel I make a ***contribution*** in my community	☐	☐	☐	☐	☐
I feel I have ***control*** in how I manage my health and wellbeing	☐	☐	☐	☐	☐
I feel I have a ***choice*** in how I manage my health and wellbeing	☐	☐	☐	☐	☐
I feel that I am part of my ***community***	☐	☐	☐	☐	☐

Valuing the healthy connections app

Now we would like you to **rate** the features of the health connections app. Using a scale of **1 (Not important) to 5 (Very important),** please rate the features in terms of importance to YOU. Please place only one tick for each of the 6 features to represent your importance rating.

		**▯-Not important ....very important▯**				
**Features**	**What do they offer?**	**1**	**2**	**3**	**4**	**5**
**Connections**	Ease connecting with others through technology	☐	☐	☐	☐	☐
**Control**	Control over your health and your care	☐	☐	☐	☐	☐
**Choice**	Awareness of the possible health and care options available to you	☐	☐	☐	☐	☐
**Collaboration**	The opportunity to talk and share decisions and experiences with others	☐	☐	☐	☐	☐
**Community**	Connecting you with your community	☐	☐	☐	☐	☐
**Contribution**	Opportunities to contribute to your local community	☐	☐	☐	☐	☐

Currently, we do not know how much people value the features of ***Healthy Connections***. One way of finding this out is to ask people to imagine how much they would be willing to pay for such a provision.

Please remember that the values we are asking should be considered as a **monthly subscription fee**. Think about how much you currently pay to stay connected to others (e.g. mobile phone charges, broadband) or for other health benefits (e.g. gym membership) and consider how much you would value the features of ***Healthy Connections*** in relation to these other monthly payments you make.

Given your current circumstances, what is the maximum amount that you would be willing to pay monthly to have access to Healthy Connections and all its 6 features?

Monthly amount £______________

Your current 'healthy connections' situation	Situation B
Your ***current*** level of connectedness with friends, family and health care providers.	You feel ***much more*** connected with friends and family and health care providers
Your ***current*** level of contribution to your community.	You make ***much more*** of a contribution to your community.
Your ***current*** feeling of control over your own health and wellbeing.	You feel ***much more*** in control over your own health and wellbeing.
Your ***current*** level of choice on how you manage your own health and wellbeing.	You have ***much more*** choice on how you manage your own health and wellbeing.
Your ***current*** level of collaboration with others in making decisions about your own health and wellbeing.	Your feel ***much more*** able to collaborate with others in making decisions about your own health and wellbeing.
Your ***current*** level of feeling part of the community.	Your feel ***much more*** part of the community.

Given your current 'healthy connections' circumstances, what is the maximum amount **per month** would you be willing to pay for a health connections app which **moved you** from **your current situation** to **Situation B** above? £____________________

Now, please consider the two following situations and think about the **maximum amount you would be willing pay per month to move from your current 'healthy connections' Situation to Situation B**. In other words, what is the maximum amount you would be willing to pay per month for an improved scenario with increased levels of connectedness, contribution, control, choice, collaboration and sense of community.

FINALLY, PLEASE TELL US A BIT ABOUT YOU

1. What is your gender?

☐Male ☐Female

2. How old are you?

☐18-25yrs

☐26-35yrs

☐36-45yrs

☐46-55yrs

☐56-65yrs

☐66-75yrs

☐76-85yrs

☐86-95yrs

☐96+yrs

3. What is your relationship status?

☐Married ☐Single ☐Cohabiting

☐Partner, not living together ☐Civil partnership ☐Widowed

☐Divorced ☐Separated but not divorced

☐Other, *please specify*..................................

4. **Do you have any children?**

☐Yes, I have ....child(ren) ☐No

5. **Do you look after, or give any support to family, friends, neighbours or others because of long term physical or mental ill health/disability or problems related to old age (do not count anything you do as part of paid employment)?**

☐No

☐ Yes, 1-19 hours a week

☐ Yes, 20-49 hours a week

☐ Yes, 50 or more hours a week

6. Are you currently employed?

☐Full-time

☐Part-time

☐ Self-employed

☐Student or training

☐Unemployed

☐Retired

☐Not working due to illness

☐ Prefer not to answer

7. **What is your highest educational qualification?**(*Please tick **one** box)*

☐No formal educational qualifications

☐O Grade, Standard Grade, GCSE, CSE, or equivalent

☐IB, Advanced Higher/A Level, Higher/AS Level, Advanced Senior cert, CSYS or equivalent

☐Apprenticeships or trade qualification

☐HNC, HND, SVQ, RSA Higher Diploma or equivalent

☐Undergraduate degree

☐Postgraduate degree

☐Other technical or business qualification / certificate

8. **Which best represents your TOTAL ANNUAL HOUSEHOLD INCOME from all sources. Do not deduct Tax, National Insurance, Health Insurance payments, or your contributions to pension schemes. Also do not count loans.**

☐Less than £14,999

☐£15,000 - £29,999

☐£30,000 - £49,999

☐£50,000 or more

9. **What is your ethnic group?**
*Please tick **one** box*

☐Scottish/English/Welsh/Northern Irish/British

☐Irish

☐Gypsy or Irish Traveller

☐Any other White background, please describe:___________________

☐White and Black Caribbean

☐White and Black African

☐White and Asian

☐Any other Mixed/Multiple ethnic background, please describe:___________________

☐Indian

☐Pakistani

☐Bangladeshi

☐Chinese

☐Any other Asian background, please describe:___________________

☐African

☐Caribbean

☐ Any other Black/African/Caribbean background, please describe:___________________

☐Arab

☐Any other ethnic group, please describe:___________________

10. **Computers (any computer including a PC, laptop, tablet, ipad)**
*Please tick **one** box*

☐ I do not own a computer

☐ I own a computer but never use it

☐ I own a computer but rarely use it

☐ I own a computer and use it regularly

☐ I own more than one computer and use them regularly

11. **Internet** *Please tick **one** box*

☐ I have no access to the Internet at home

☐ I have access to the Internet at home but never use it

☐ I have access to the Internet at home but rarely use it

☐ I have access to the Internet at home and use it regularly

12. **Smartphones (a mobile phone which you can use for email, browsing the internet, downloading apps etc)**
*Please tick **one** box*

☐ I do not own a smartphone

☐ I own a smartphone but never use it

☐ I own a smartphone but rarely use it

☐ I own a smartphone and use it regularly

13. **What is the total monthly amount you spend on all your phone, internet and any additional features (app subscriptions)**
*Please tick **one** box*

☐ £0-10

☐ £11-20

☐ £21-30

☐ £31-40

☐ £41+

14. **Have you ever used an app for improving your fitness/health or wellbeing?**
*Please tick **one** box*

☐ No

☐ Yes

If ***Yes***, please answer **Q15** and **Q16**. If ***No***, go straight to **Q17**.

15. **Please state the name of app(s) and the total cost of the app(s)**:

__________________ £____________

__________________ £____________

__________________ £____________

16. **Since using this app**

(insert name of most recent app): _____________________

*How do you feel it has changed your health? Please tick **one** box*

☐ I feel much more healthy

☐ I feel more healthy

☐ No change

☐ I feel less healthy

☐ I feel much less healthy

17. In general, would you say your health is…?
Please tick one box

☐Excellent

☐Very good

☐Good

☐Fair

☐Poor

18. **Do you have any long term conditions (e.g. asthma, diabetes, cancer, psoriasis, lung disease, heart disease, depression etc)?**

☐No

☐Yes

If ***Yes,*** please tick the box that correctly indicates the number of conditions you suffer from:

☐1

☐2

☐3

☐4

☐5

☐6 or more____

19. Do you take any medications regularly?

☐No

☐Yes

If ***Yes,*** please tick one box that matches how many different medications you currently take each day:

☐1

☐2

☐3

☐4

☐5

☐6

☐7

☐8

☐9

☐10 or more__________

20.Do you currently smoke ?
☐No
☐Yes

21. **How often do you have a drink containing alcohol?**

*Please tick **one** box*

☐ Never

☐ Monthly or less

☐2-4 times a month

☐2-3 times a week

☐ 4 or more times a week

22. **How important is staying healthy to you?**
*Please tick **one** box*

☐ Very Important

☐ Somewhat important

☐ Irrelevant to me
