# Supplementary material for: Valuing Mobile Health: An Open-Ended Contingent Valuation Survey of a National Digital Health Program
Source: JMIR Mhealth Uhealth. 2019 Jan 17;7(1):e3. doi: 10.2196/mhealth.9990 (PMC6354197; doi:10.2196/mhealth.9990)
Supplement: Multimedia Appendix 2 [file mhealth_v7i1e3_app2.pdf]

Multimedia appendix B

Healthy Connections UK general population sample statistics

Socio-demographic details

Number of respondents	1697	
Variables	No. respondents	%
Gender		
Male	826	49
Female	871	51
Age		
18-25	271	16 %
26-35	271	16 %
36-45	306	18 %
46-55	271	16 %
56-65	238	14 %
65+	340	20 %
Relationship Status		
Married	888	52
Single	375	22
Co-habiting	203	12
Partner, not living together	54	3
Civil Partnership	12	1
Widowed	48	3
Divorced	92	5
Separated but not divorced	23	2
Other, please specify	2	0
Do you have children?		
Yes	1044	62
No	653	38
Do you look after, or give support to family, friends, neighbours or others because of long-term physical or mental ill health/disability or problems related to old age		
No	1287	76
Yes, 1-19 hours a week	279	16
Yes, 20-49 hours a week	84	5
Yes, 50 or more hours a week	47	3
Employment status		
Full-time	671	40
Part-time	218	13
Self-employed	98	6
Student or training	80	5
Unemployed	120	7
Retired	427	25
Not working due to illness	66	3
Prefer not to answer	17	1

Highest educational qualification		
No formal educational qualifications	76	4
O Grade, Standard Grade, GCSE, CSE, or equivalent	319	19
IB, Advanced Higher/A Level, Higher/AS Level, Advanced Senior cert, CSYS or equivalent	263	16
Apprenticeships or trade qualification	105	6
HNC, HND, SVQ, RSA Higher Diploma or equivalent	208	12
Undergraduate degree	409	24
Postgraduate degree	244	15
Other technical or business qualification / certificate	73	4
Total Household Income		
Less than £14,999	317	19
£15,000 - £29,999	516	31
£30,000 - £49,999	501	29
£50,000 or more	363	21
Ethnicity		
Scottish/English/Welsh/Northern Irish/British	1421	84
Irish	20	2
Gypsy or Irish Traveller	3	0
Any other White background, please describe:	69	4
White and Black Caribbean	13	1
White and Black African	6	0
White and Asian	22	1
Any other Mixed/Multiple ethnic background, please describe:	6	0
Indian	36	2
Pakistani	17	1
Bangladeshi	15	1
Chinese	18	1
Any other Asian background, please describe:	11	1
African	14	1
Caribbean	12	1
Any other Black/African/Caribbean background, please describe:	3	0
Arab	2	0
Any other ethnic group, please describe:	9	0
Computers (any computer including a PC, laptop, tablet or ipad)		
I do not own a computer	59	4
I own a computer but never use it	23	1
I own a computer but rarely use it	91	5
I own a computer and use it regularly	848	50
I own more than one computer and use them regularly	676	40
Internet		
I have no access to the Internet at home	41	2
I have access to the Internet at home but never use it	14	1
I have access to the Internet at home but rarely use it	50	3
I have access to the Internet at home and use it regularly	1592	94
Smartphones (a mobile phone which you can use for email, browsing the internet, downloading apps etc)		
I do not own a smartphone	277	16
I own a smartphone but never use it	23	1
I own a smartphone but rarely use it	131	8
I own a smartphone and use it regularly	1266	75
Total monthly amount spent on phone/internet/additional features		
£0-10	312	18

£11-20	332	20
£21-30	404	24
£31-40	306	18
£41+	343	20
Have you used an app for improving your fitness/health or wellbeing?		
No	1136	67
Yes	561	33
General Health		
Poor	106	6
Fair	318	19
Good	664	39
Very good	481	28
Excellent	128	8
Long-term health conditions		
No	1066	63
Yes	631	37
If yes, number of conditions		
1	292	46
2	177	28
3	82	13
4	43	7
5	25	4
6 or more	12	2
Take medications regularly		
No	890	52
Yes	807	48
If yes, number of medications		
1	212	26
2	190	24
3	129	16
4	98	12
5	58	7
6	47	6
7	20	2
8	13	2
9	9	1
10 or more	31	4
Smoke		
No	1451	85
Yes	246	15
How often you drink alcohol		
Never	290	17
Monthly or less	397	23
2-4 times a month	415	24
2-3 times a week	364	22
4 or more times a week	231	14
How important is staying healthy?		
Irrelevant to me	27	2
Somewhat important	544	32
Very Important	1126	66

Healthy Connections ‘dallas-like’ sample statistics

Socio-demographic details

Number of respondents	305	
Variables	No. respondents	%
Gender		
Male	85	28
Female	220	72
Age		
0-50	149	49
40-55	30	10
50-75	114	37
75-140	12	4
Relationship Status		
Married	158	52
Single	63	21
Co-habiting	36	12
Partner, not living together	8	2
Civil Partnership	3	1
Widowed	13	4
Divorced	21	7
Separated but not divorced	2	1
Other, please specify	1	0
Do you have children?		
Yes	193	63
No	112	37
Do you look after, or give support to family, friends, neighbours or others because of long-term physical or mental ill health/disability or problems related to old age		
No	212	70
Yes, 1-19 hours a week	72	23
Yes, 20-49 hours a week	9	3
Yes, 50 or more hours a week	12	4
Employment status		
Full-time	121	40
Part-time	46	15
Self-employed	15	5
Student or training	13	4
Unemployed	16	5
Retired	76	25
Not working due to illness	14	5
Prefer not to answer	4	1
Highest educational qualification		
No formal educational qualifications	15	5
O Grade, Standard Grade, GCSE, CSE, or equivalent	57	19
IB, Advanced Higher/A Level, Higher/AS Level, Advanced Senior cert, CSYS or equivalent	33	11
Apprenticeships or trade qualification	15	5
HNC, HND, SVQ, RSA Higher Diploma or equivalent	41	13
Undergraduate degree	89	29

Postgraduate degree	42	14
Other technical or business qualification / certificate	13	4
Total Household Income		
Less than £14,999	46	15
£15,000 - £29,999	102	33
£30,000 - £49,999	81	27
£50,000 or more	76	25
Ethnicity		
Scottish/English/Welsh/Northern Irish/British	263	85
Irish	4	2
Gypsy or Irish Traveller	1	0
Any other White background, please describe:	10	3
White and Black Caribbean	1	0
White and Black African	1	0
White and Asian	4	2
Any other Mixed/Multiple ethnic background, please describe:	1	0
Indian	3	2
Pakistani	3	2
Bangladeshi	1	0
Chinese	4	2
Any other Asian background, please describe:	1	0
African	1	0
Caribbean	2	0
Any other Black/African/Caribbean background, please describe:	0	0
Arab	0	0
Any other ethnic group, please describe:	5	2
Computers (any computer including a PC, laptop, tablet or ipad)		
I do not own a computer	8	3
I own a computer but never use it	2	1
I own a computer but rarely use it	13	4
I own a computer and use it regularly	144	47
I own more than one computer and use them regularly	138	45
Internet		
I have no access to the Internet at home	5	2
I have access to the Internet at home but never use it	2	1
I have access to the Internet at home but rarely use it	10	3
I have access to the Internet at home and use it regularly	288	94
Smartphones (a mobile phone which you can use for email, browsing the internet, downloading apps etc)		
I do not own a smartphone	43	14
I own a smartphone but never use it	3	1
I own a smartphone but rarely use it	24	8
I own a smartphone and use it regularly	235	77
Total monthly amount spent on phone/internet/additional features		
£0-10	61	20
£11-20	67	22
£21-30	52	17
£31-40	70	23
£41+	55	18
Have you used an app for improving your fitness/health or wellbeing?		
No	194	64

Yes	111	36
General Health		
Poor	10	3
Fair	52	17
Good	115	38
Very good	95	31
Excellent	33	11
Long-term health conditions		
No	199	65
Yes	106	35
If yes, number of conditions		
1	56	53
2	30	28
3	14	13
4	4	4
5	2	2
6 or more	0	0
Take medications regularly		
No	172	56
Yes	133	44
If yes, number of medications		
1	40	30
2	29	22
3	20	15
4	18	14
5	13	10
6	7	5
7	3	3
8	1	0
9	0	
10 or more	2	1
Smoke		
No	261	86
Yes	44	14
How often you drink alcohol		
Never	50	17
Monthly or less	62	20
2-4 times a month	63	21
2-3 times a week	77	25
4 or more times a week	53	17
How important is staying healthy?		
Irrelevant to me	8	3
Somewhat important	90	30
Very Important	207	67
