# Supplementary material for: Valuing Mobile Health: An Open-Ended Contingent Valuation Survey of a National Digital Health Program
Source: JMIR Mhealth Uhealth. 2019 Jan 17;7(1):e3. doi: 10.2196/mhealth.9990 (PMC6354197; doi:10.2196/mhealth.9990)
Supplement: Multimedia Appendix 3 [file mhealth_v7i1e3_app3.pdf]

Multimedia appendix C

Cohorts Socio-demographic and economic characteristics and WTP linear regressions (Adjusted for age, total household income and gender)

Variable	General UK population (n=1697)				Dallas-like (n=305)			
	Coef.	P-value	95% CI		Coef.	P-value	95% CI	
**Age**	-0.1	0.00	-0.02	-0.01	-0.02	0.00	-0.03	-0.01
**Gender male (REF)**	-	-	-	-	-	-	-	-
**Female**	-0.02	0.73	-0.16	0.11	-0.35	0.04	-0.69	-0.01
**Which best represents your TOTAL ANNUAL HOUSEHOLD INCOME from all sources. Do not deduct Tax, National Insurance, Health Insurance payments, or your contributions to pension schemes. Also do not count loans.**								
**Less than £14,999**	-	-	-	-	-	-	-	-
**£15,000 - £29,999**	0.21	0.04	0.14	0.4	0.29	0.22	-0.17	0.76
**£30,000 - £49,999**	0.18	0.08	-0.02	0.38	0.11	0.65	-0.37	0.6
**£50,000 or more**	0.1	0.37	-0.11	0.31	0.03	0.9	-0.47	0.54
**In general, would you say your health is...?**								
**Poor**	-	-	-	-	-	-	-	-
**Fair**	0.07	0.64	-0.23	0.37	0.58	0.2	-0.31	1.47
**Good**	0.15	0.32	-0.14	0.44	0.79	0.07	-0.06	1.65
**Very good**	0.38	0.01	0.08	0.69	0.90	0.04	0.03	1.78
**Excellent**	0.64	0.00	0.27	1.01	1.27	0.01	0.32	2.23
**Do you have any long term conditions?**								
**No**	-	-	-	-	-	-	-	-
**Yes**	-0.04	0.61	-0.2	0.12	0.07	0.72	-0.32	0.47
**Do you take medications regularly?**								
**No**	-	-	-	-	-	-	-	-
**Yes**	0.16	0.05	-0.01	0.32	0.08	0.7	-0.31	0.47

Cohorts familiarity and accessibility to mhealth and technology and WTP linear regressions (Adjusted for age, total household income and gender)

Variable	General UK population (n=1697)				Dallas-like (n=305)			
	Coef.	P-value	95% CI		Coef.	P-value	95% CI	
**Computers (any computer including PC, laptop, tablet, ipad)**								
**I do not own one**	-	-	-	-	-	-	-	-
**I own one but never use it**	0.19	0.58	-0.47	0.84	-0.91	0.39	-2.98	1.16
**I own one but rarely use it**	-0.5	0.03	-0.95	-0.05	0.12	0.83	-1.03	1.28
**I own one and use it regularly**	-0.17	0.36	-0.54	0.2	-0.10	0.83	-1.04	0.83
**I own more than one and use them regularly**	-0.22	0.27	-0.6	0.17	0.23	0.63	-0.71	1.18
**Internet**								
**I have no access at home**	-	-	-	-	-	-	-	-
**I have access at home but never use it**	1.18	0.01	0.34	2.01	1.12	0.35	-1.21	3.46
**I have access at home but rarely use it**	0.39	0.17	-0.17	0.96	-0.44	0.55	-1.91	1.02
**I have access at home and use it regularly**	-0.50	0.02	-0.93	-0.07	-0.76	0.24	-2.03	0.5

**Smartphones (a mobile phone which you can use for email, browsing the internet, downloading apps etc)**								
**I do not own one**	-	-	-	-	-	-	-	-
**I own one but never use it**	0.24	0.43	-0.35	0.83	-0.16	0.85	-1.82	1.51
**I own one but rarely use it**	0.22	0.12	-0.06	0.51	0.31	0.35	-0.35	0.98
**I own one and use it regularly**	-0.02	0.84	-0.22	0.18	0.08	0.76	-0.43	0.59
**Total monthly amount spent on all your phone, internet and any additional features**								
**£0-10**	-	-	-	-	-	-	-	-
**£11-20**	0.07	0.52	-0.14	0.28	0.33	0.16	-0.14	0.8
**£21-30**	0.34	0.01	0.14	0.54	0.001	0.99	-0.51	0.51
**£31-40**	0.46	0.00	0.24	0.68	0.34	0.17	-0.15	0.82
**£41+**	0.52	0.00	0.30	0.74	0.06	0.80	-0.44	0.57
**Have you used an app for improving your fitness/health or wellbeing?**								
**No**	-	-	-	-	-	-	-	-
**Yes**	0.01	0.95	-0.24	0.26	0.26	0.39	-0.33	0.85
**Number of health apps used previously**								
**Number of health apps used previously**	0.06	0.46	0.09	0.20	-0.22	0.21	-0.57	0.13
**How much spent on health apps previously**								
**Cost (£)**	0.01	0.01	0.01	0.01	0.01	0.00	0.01	0.02
